# A Real-Time Multi-Class Human Activity Monitoring System Using mmWave Radar

**DOI:** 10.3390/s26103145

**Published:** 2026-05-15

**Authors:** Doheon Kim, Sol Lee, Myeongjin Lee

**Affiliations:** 1 School of Electronics and Information Engineering, Korea Aerospace University, Goyang 10540, Gyeonggi, Republic of Korea; doo3003@kau.kr (D.K.); ekmonet1@kau.kr (S.L.); 2 Department of Smart Air Mobility, Korea Aerospace University, Goyang 10540, Gyeonggi, Republic of Korea

**Keywords:** human activity recognition, range–Doppler map, mmWave radar, online activity monitoring

## Abstract

This paper presents a robust and efficient mmWave radar-based human activity recognition (HAR) framework optimized for practical real-time indoor deployment. Addressing computational inefficiencies and limited recognition scopes in existing systems, the framework introduces two core contributions: Multi-class Spatio-Temporal Network (MuST-Net), a lightweight, multi-class network, and an online detection process for enhanced temporal stability. MuST-Net utilizes a hybrid 2D convolutional neural network and temporal convolutional network architecture to recognize seven distinct classes, significantly broadening the system’s recognition repertoire. The online detection process implements a novel sliding-window post-processing chain that employs an activity-buffering mechanism, which maintains temporal continuity and effectively suppresses spurious detections at activity boundaries. Experimental results demonstrate the superior performance of our unified framework, attaining over 98.6% accuracy for multi-class classification by MuST-Net and achieving at least 97% accuracy for activity detection and a crucial 100% recall for *fall* detection. Robustness is validated across three distinct indoor environments and nine subjects—with two of the three sites entirely unseen during training—confirming strong generalization under installation, environment, and subject variations.

## 1. Introduction

With the rise of an aging society, vulnerable groups like single-person elderly households face difficulties requesting immediate assistance during indoor falls. Additionally, the need for indoor activity monitoring is growing for home-care patients and those spending extended periods indoors due to the spread of remote work. Consequently, systems are being actively developed to continuously observe subjects in a contactless manner, enabling early detection of abnormal conditions and monitoring of indoor activity levels.

Extensive research has been conducted on Human Activity Recognition (HAR) using various sensors, including wearables [1,2], vision systems [3,4], LiDAR [5,6], ultrasonic sensors [7,8], and radar [9,10,11,12,13,14]. However, wearables require continuous attachment, while ultrasonic and vision-based systems face limitations in range, performance variability, and privacy. Although LiDAR offers high precision, it remains costly and sensitive to environmental conditions. In contrast, radar technology provides a wider detection range, cost-effectiveness, and environmental robustness. For confined personal spaces like studio apartments, which require a balance of accuracy, real-time responsiveness, and privacy, mmWave FMCW radar-based HAR emerges as a promising alternative to vision-based approaches.

Despite significant advances, existing mmWave-based HAR systems face practical limitations in monitoring scope and operational continuity. First, some highly efficient systems, such as the Range–Doppler–Time Network (RDTNet), are primarily optimized for binary fall detection within a two-stage framework [12]. These systems typically remain in a dormant state until triggered by specific movement thresholds such as a barycenter descent. Although this reduces power consumption, such a trigger-based approach inherently limits the capability for integrated, continuous monitoring of various daily activities. Second, while certain multi-class frameworks, such as MFECNet [14], expand the recognition repertoire to multiple activities, they often rely on multi-feature fusion (e.g., integrating range–time and Doppler–time maps). This multi-stream processing approach demands significantly more computational resources, posing a major hurdle for deployment on resource-constrained embedded edge devices that require a balance between high accuracy and real-time responsiveness. Third, some studies rely on traditional machine learning without temporal sequence modeling. For example, Liang et al. utilized random forest and three-layer BP neural networks for binary classification (fall vs. standing) [15]. Their system requires complex preprocessing to generate point-cloud data and lacks the ability to capture dynamic temporal patterns necessary for complex multi-class recognition.

Furthermore, many existing frameworks suffer from temporal discontinuity due to their reliance on fixed-stride sequence partitioning for classification [10,14,16]. This static window-based approach frequently induces false detections at activity boundaries and decision delays. Without a dedicated mechanism to handle the transitions between successive actions in a live stream, these systems struggle to maintain the stability required for reliable real-time monitoring. For example, Scherer et al. [16] proposed a lightweight convolutional neural network (CNN) utilizing spatio-temporal features, but the fixed-window design limited continuous activity recognition. Similarly, Kittiyanpunya et al. [10] developed an LSTM-based fall detection model, but stride-based processing inducing false positives in the boundary domain continued.

Existing work highlights an important gap with respect to the inability to achieve broad coverage of activities without sacrificing real-time efficiency and temporal stability. To improve mmWave-based monitoring, it is essential to bridge the performance of high-overhead, multi-class models with the efficiency of specialized binary detectors. Furthermore, to overcome boundary-induced errors, we need to shift from static segmentation to a framework that handles streaming radar signatures. Addressing these challenges is essential to move beyond sporadic event detection to autonomous and robust indoor health monitoring.

To this end, we propose a lightweight, real-time HAR framework integrating a CNN for spatial feature extraction from range–Doppler radar data and a Temporal Convolutional Network (TCN) for effective modeling of temporal patterns. This hybrid MuST-Net architecture enables robust multi-class action recognition. Crucially, a subsequent process is applied to ensure temporal consistency by buffering recent predictions and confirming actions only after sustained persistence, effectively filtering transient noise and reducing double counting with low computational overhead.

The key contributions of our research are summarized as follows.

1. Lightweight multi-class model: We extend the binary fall-detection structure [12] into a multi-class network called MuST-Net, which can simultaneously distinguish among six actions and a state of no action. MuST-Net reduces representation loss by preserving the initial resolution of RDMs and gradually downsampling through CNN layers. Unnecessary parameters and operations are removed via selective average pooling and kernel design. The model stably learns short- and long-term temporal patterns by tuning the depth and receptive fields of the TCN.
2. Activity detection process: A robust event-level activity detection process is proposed that verifies multi-class activity classification outputs from MuST-Net across time, confirming final activity labels by examining temporal consistency. This framework enables real-time detection and counting for each activity, thereby supporting comprehensive monitoring and logging of indoor behavior.
3. Open-environment generalization: Diverse actions in actual living spaces are analyzed, and randomness in location, orientation, and subject is incorporated into labeling to ensure robust generalization. Specifically, walking actions are divided into forward and backward, while classes such as *sit*, *stand*, *squat*, and *fall* contain variations in orientation, distance, and position to maximize intra-class diversity. With this approach, the system remains robust under distribution shifts such as changes in installation locations, changes in furniture or obstacles, and variations in action positions, thereby mitigating environmental and subject dependence. This generalization is validated through online detection experiments conducted across three distinct indoor environments (Sites A, B, and C) with nine subjects, where the MuST-Net model was trained exclusively on Site A data.

The remainder of this paper is organized as follows. Section 2 details the methodology, including the radar configuration and RDM data collection for seven activity classes. Section 3 introduces the MuST-Net architecture, a hybrid CNN-TCN designed for continuous real-time recognition. Section 4 evaluates its performance and real-time generalization capabilities, while Section 5 presents the conclusions and discusses future work.

## 2. Data Collection and System Configuration

### 2.1. Data Collection Environment

**Figure 1:**
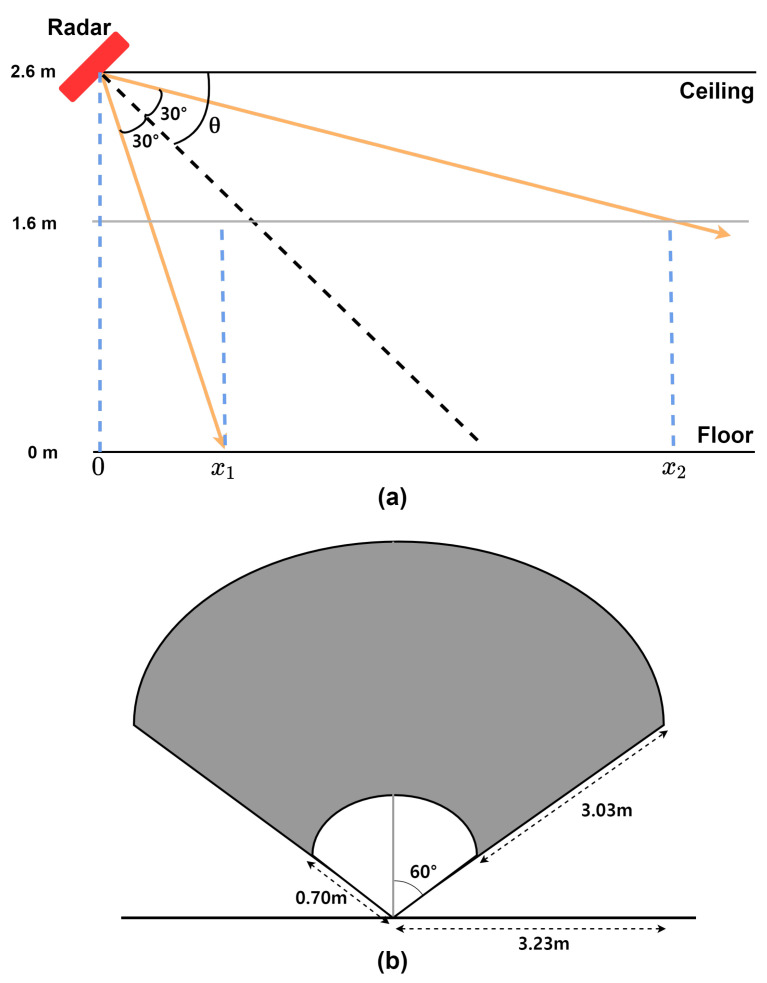
Radar Installation: (**a**) radar installation angle; (**b**) horizontal beam width and detection range.

We employ the TI AWR1843 radar to enable real-time processing of Range–Doppler Maps (RDMs), as summarized in Table 1. Figure 1 illustrates the installation geometry: the sensor is mounted at the ceiling height, i.e., h=2.6 m, with a downward tilt of $\theta =45^{\circ }$ relative to the floor. Half the vertical field of view of the radar is $\phi =30^{\circ }$; in a vertical cross-section, the lower and upper boundary rays have slopes of tan(θ+ϕ) and tan(θ−ϕ). Let *x* denote the distance projected on the floor from the radar footprint and *H* denote the height of the human body, fixed at 1.6 m in this study. The full body lies entirely within the FoV when

(1) $$x_{1}=\frac{h}{\tan(\theta +\phi )},\;x_{2}=\frac{h-H}{\tan(\theta -\phi )},$$

so the admissible distance is $x\in [x_{1},x_{2}]$. A necessary condition for this interval to exist is

(2) $$f\left(\theta \right)=\frac{x_{1}}{x_{2}}=\frac{h\;\tan(\theta -\phi )}{\left(h-H)\;\tan(\theta +\phi \right)}\leq 1.$$

Numerically, substituting h=2.6 m, H=1.6 m, $\theta =45^{\circ }$, and $\phi =30^{\circ }$ gives $x_{1}\approx 0.70\;m$ and $x_{2}\approx 3.73\;m$. Hence, whole-body observation is feasible for floor-projected distances between 0.70 and 3.73 m in front of the radar.

**Figure 2:**
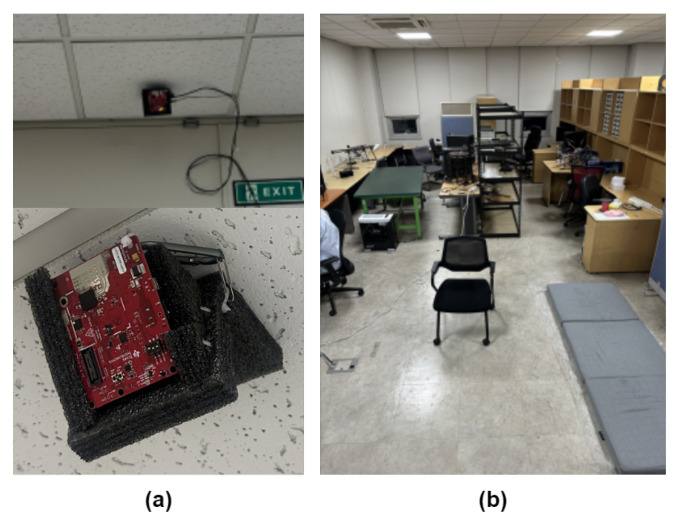
Experimental environment: (**a**) radar installation; (**b**) indoor environment (Site A).

Combining this valid range with the horizontal aperture shown in Figure 1 yields a theoretical floor-projected detection area of approximately $14.06\;m^{2}$. This coverage adequately covers the core living area of typical single-occupancy dwellings, aligning with international housing standards [17,18,19]. In practice, as illustrated in Figure 2, subjects performed actions within a 5 × 5 m^2^ region in front of the radar. The ceiling-mounted configuration adopted here differs from the horizontal chest-height placement used in some prior works [20,21], where subjects face the sensor’s line of sight and micro-Doppler spectrograms are used as input. In contrast, our system uses range–Doppler maps in an unconstrained indoor setting where subjects move freely without any requirement to face the sensor. The overhead geometry captures motion signatures, regardless of the subject’s facing direction, and provides symmetric wide-area floor coverage, making it well-suited to this deployment scenario. This installation approach is consistent with the most directly comparable state-of-the-art systems, including RDTNet [12] and MFECNet [14], both of which independently adopt ceiling-mounted configurations at an approximately 3 m height with RDM inputs. Yang and Ye [12] explicitly note that ceiling deployment is advantageous over horizontal placement for this class of systems.

### 2.2. Target Activities: Detection Classes

Six indoor actions—*fall*, *sit*, *stand*, *walk-toward (walkT)*, *walk-away (walkA)*, and *squat*—were selected as core classes for radar-based activity recognition. The selection reflects a focus on motion primitives and posture transitions commonly observed in daily living environments, which are central to the monitoring of functional health. However, *fall* is specifically included to address the increased risk faced by older people and those living alone, making it essential for incident detection. *Squat* is also chosen, given its role as composite movement that integrates elements of both sitting and standing, offering richer indicators for health assessment in indoor settings.

**Figure 3:**
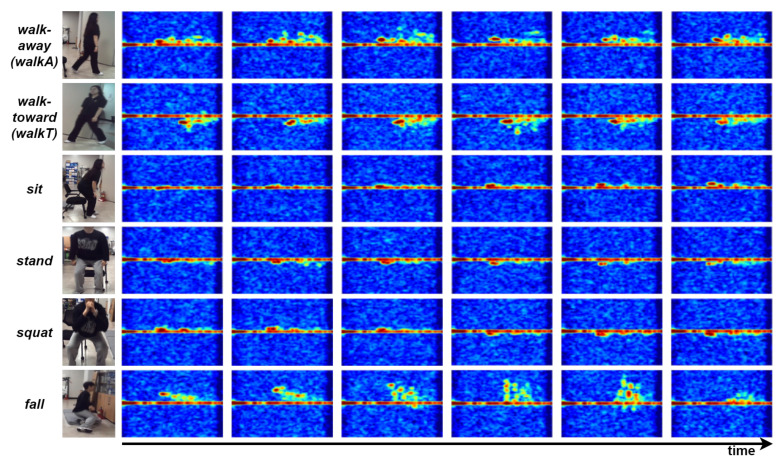
RDM sequences by action. The colors in the RDMs indicate the relative radar signal intensity, and the corresponding color scale is shown in Figure 4.

Figure 3 presents time-evolving RDM sequences and video reference frames for each action class, allowing for direct visualization of distinctive spatial and temporal radar signatures. Dynamic activities such as *walk-toward* and *walk-away* feature prominent Doppler shifts with clear directional contrasts, whereas more static actions like sitting and standing show stable patterns near zero Doppler frequency. *Squat* yields mixed characteristics, combining features of seated and upright postures, and *fall* is marked by abrupt and irregular signal changes, highlighting its importance for automatic risk detection.

These observations confirm that each movement type exhibits unique radar-based spatial–temporal features, which can be leveraged for accurate activity classification and reliable monitoring. Thus, the RDM sequence not only clarifies the basis for class selection but also underscores the importance of such classes in practical health and safety monitoring scenarios.

### 2.3. Data Collection and Processing

#### 2.3.1. Data Collection Procedure

The input of the system is the RDM sequence that represents the time-varying distance and velocity of a person. The TI AWR1843 radar generates an RDM using a 2D signal processing chain on digitized FMCW echoes. The process starts with a 1D Fast Fourier Transform (Range-FFT) on each chirp’s samples. This resolves the beat frequency into range profiles that determine the target distance. Next, a 2D FFT (Doppler-FFT) is performed along the chirp axis for each range bin. This second FFT resolves the phase change to calculate the Doppler frequency and, thus, the target’s velocity. The magnitude of this final 2D matrix is the RDM, which serves as the sole input to the proposed recognition framework. While the radar hardware additionally supports angle-of-arrival (AoA) estimation as a downstream capability, AoA is not utilized in this work.

The range and velocity characteristics of the target activities detectable within our specified indoor environment were analyzed during mmWave radar configuration, allowing us to determine the required range and velocity limits, as well as their corresponding resolutions.

According to biomechanical studies of human movement [22,23,24], the maximum velocities for target activities (e.g., falls, walking, and squats) typically range within ±4 m/s. Specifically, impact velocities from falls are reported between 0.95 m/s and 3.40 m/s [22,23], while indoor walking and transition speeds generally remain below this threshold [24]. Thus, the velocity limit of our radar configuration was set at ±4 m/s to capture the full range of these actions.

**Figure 4:**
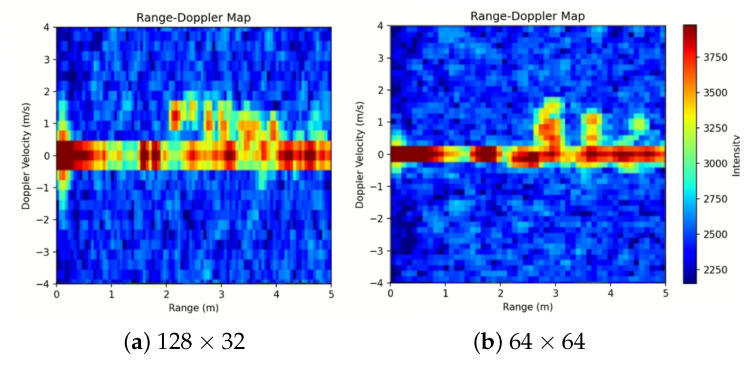
RDM resolution comparison of *fall*.

Figure 4 compares the RDMs generated with resolution configurations of 64 × 64 and 128 × 32 for the same action, serving as candidates to meet the link bandwidth constraint. While 128 × 32 provides a finer range resolution, the Doppler axis is sparse, making velocity variations blur. On the other hand, 64 × 64 secures twice the velocity resolution, making subtle velocity variations clearer with time. Considering that our key decision criterion focuses on *the velocity distribution pattern* rather than the absolute position, the higher velocity resolution of 64 × 64 provides more informative features. Transmission of a 64 × 64 RDM at 8 frames per second (FPS) requires approximately 65,536 bytes/s, utilizing 71.1% of the available 921,600 baud UART bandwidth to ensure stable real-time operations.

The waveform and FFT configuration set the unambiguous range to 5 m, resulting in a range resolution of 7.8 cm. The maximum radial velocity is ±3.95 m/s, and the corresponding Doppler resolution is 0.13 m/s. These settings are sufficient to address the high-velocity Doppler components upon falling and abrupt deceleration at impact and to maintain the time–velocity structure required to distinguish the six actions.

#### 2.3.2. Dataset Preparation

Data were collected at multiple reference points (1.2 m to 2.8 m) and orientations to ensure intra-class diversity and account for multipath interference, as detailed in Section 2.1.

**Figure 5:**
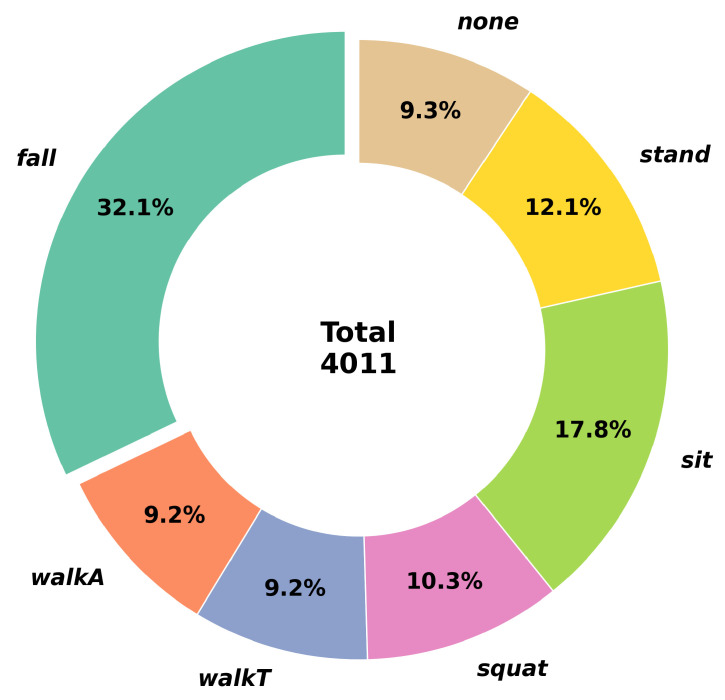
Dataset for indoor activity recognition.

Real-time RDM sequences were captured at 8 frames per second as subjects performed six different actions during each trial. Each trial lasted approximately 52 s, generating roughly 416 RDM frames. Frame-level annotations were used to accurately mark action boundaries, providing ground-truth labels for supervision. Fixed-length segments of 24 consecutive frames corresponding to 3 s segments were extracted from the annotated data, with each segment associated with its respective action label. The total dataset comprised 4011 action segments, which were divided into training and validation sets in a 70:30 ratio as shown in Figure 5.

Since the primary objective of this system involves safety-critical monitoring, our dataset is intentionally constructed with a greater proportion of *fall* instances (32.1%). This ensures the model achieves high sensitivity and robustness against various fall patterns, which typically exhibit higher intra-class variance compared to routine daily activities such as walking or sitting. While this distribution does not reflect the naturalistic rate of falls in daily life, we intentionally prioritized the collection of *fall* data to minimize false negatives in critical safety scenarios. The online detection experiments in Section 4.3, conducted on continuous unconstrained activity sequences in approximately 5 min trials across nine subjects and three sites, provide a more realistic estimate of deployment performance. To mitigate the effects of class imbalance during training, we employed a weighted cross-entropy loss function. To define the *none* class, we extracted segments from 52 s sequences in which no specific activity was performed—specifically, the transition periods between distinct actions—and labeled them as training data.

Six participants (four males and two females) participated in data collection, completing repeated trials for each action. Their heights ranged from 1.60 to 1.80 m. All RDM segments were stored in a consistent numeric type and normalized for training stability. Normalization statistics (mean and standard deviation) were computed exclusively on the training split, then applied unchanged to the validation split (and to a test split when used) to prevent leakage and support generalization.

## 3. Proposed Monitoring Framework

### 3.1. System Overview

**Figure 6:**
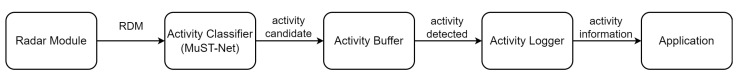
Overview of the real-time indoor activity monitoring system.

As illustrated in Figure 6, the proposed mmWave radar-based HAR framework provides a robust and contactless solution for continuous indoor activity monitoring. The system captures 64 × 64 RDMs at 8 FPS, prioritizing a 0.13 m/s velocity resolution to effectively distinguish the subtle motion variations required for accurate classification.

The core processing pipeline involves three integrated steps designed for continuous monitoring, as detailed in the following subsections:

1. Spatio-Temporal Feature Extraction (Section 3.2): The input RDM sequence feeds into the proposed MuST-Net. This lightweight hybrid architecture extracts both spatial features from individual RDM frames via a CNN module and temporal dynamics from the sequence via a TCN module.
2. Activity Detection (Section 3.3): The per-frame predictions are stabilized into reliable event-level outputs with frame-level predictions using a composite activity-confirming framework on the MuST-Net output stream. An activity buffer confirms activities only when an activity candidate persists with high confidence over a set period, ensuring accurate detection. The algorithm logs indoor activities by counting occurrences based on the type of behavior.

### 3.2. MuST-Net: Multi-Class Activity Classification

The development of deep learning models for radar-based HAR has widely utilized CNNs, employing 1D, 2D, or 3D convolution methods based on the characteristics of input data. Although 3D convolutions are proficient in jointly learning spatio-temporal features from the full Range–Doppler–Time cube, this capability incurs a significantly higher computational cost, increased network parameters, and complex storage demands, thereby hindering real-time deployment on resource-constrained embedded devices.

Our design is based on RDTNet [12], which uses a hybrid 2D-CNN/TCN structure to achieve high efficiency. Employing parameter sharing between 2D-CNN modules for spatial feature extraction, this architecture significantly reduces the computational burden compared to full 3D-CNN models such as 3DTSNet [13].

**Figure 7:**
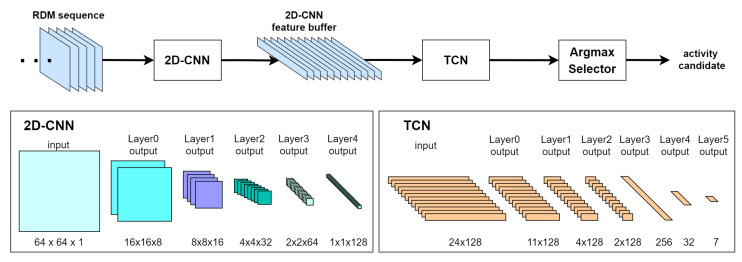
MuST-Net architecture.

However, RDTNet is primarily limited by its binary classification focus and its dependence on an initial barycenter trigger for input screening. To overcome these constraints, we propose MuST-Net in Figure 7, which extends these capabilities for the uninterrupted, multi-class monitoring of diverse indoor activities without the need for preliminary thresholds.

MuST-Net is a lightweight hybrid architecture designed to broaden the capabilities of radar-based HAR for robust online monitoring of diverse activities in indoor environments. The critical enhancements in MuST-Net over RDTNet are outlined as follows:

1. Multi-Class Expansion: MuST-Net extends the recognition capability from binary fall detection to simultaneously recognize seven distinct classes: *fall*, *walk-away*, *walk-toward*, *squat*, *sit*, *stand*, and *none*. This approach addresses the limitations of existing research that focuses on a limited set of actions.
2. Full Field Coverage: MuST-Net removes the prerequisite input thresholding employed by RDTNet. Instead, it processes the RDM data derived from the entire radar coverage. This enables the network to effectively capture diverse motions across the full radar field, which is essential for accurate multi-class classification.

MuST-Net retains the hybrid spatial–temporal extraction concept while prioritizing a low parameter count and multi-class precision for on-device deployment. Detailed network configurations are specified in Table 2a,b. This allows the system to continuously acquire 64 × 64 RDMs at 8 FPS, extracting spatial features via the CNN and subsequently modeling temporal dynamics via the TCN, resulting in a model that is lighter than a full 3D processing pipeline.

The network design prioritizes low parameter count and high accuracy for embedded deployment:

1. CNN Module (Spatial Feature Extraction): This module extracts spatial features from the 64 × 64 RDM frame at a specific timestamp. The model reduces representation loss by preserving the initial resolution of the RDMs and gradually downsampling through CNN layers. Efficiency is achieved by utilizing selective average pooling and carefully designed kernels to remove unnecessary parameters and operations.
2. TCN Module (Temporal Classification): The frame-level spatial features are organized along the time axis and fed into the TCN module. The TCN uses group convolutions (3 × 1, with 4 × 1 in the first layer) and pointwise convolutions (1 × 1). Its depth and receptive fields are tuned to stably learn both short-term and long-term temporal patterns.
3. Classification Output: Unlike binary classifiers (like RDTNet) that utilize a sigmoid function, MuST-Net uses a softmax function in the output layer to provide multi-class probability outputs. Compared to RDTNet [12], MuST-Net adopts a resolution-preserving CNN with selective average pooling and a narrower grouped, pointwise TCN, resulting in a more compact overall design, while removing the trigger-based input gating to enable uninterrupted multi-class monitoring.

For real-time inference from the online stream of RDMs from the radar module, the system processes a sliding window with a length of L=24 frames. This window captures the RDMs ($\left\{x_{t-L+1},\ldots ,x_{t}\right\}$) for input to the TCN. The TCN gives the prediction (${\hat{y}}_{t}=\mathrm{TCN}\left(x_{t-L+1:t}\right)$) and its probability ($p_{t}$). Except for the initial warm-up period, where t<L, the predictions update every frame. These per-frame outputs (${\hat{y}}_{t},p_{t}$) are subsequently fed to the post-processing chain for event-level decisions, which are detailed in Section 3.3.

### 3.3. Online Activity Detection and Post-Processing

**Figure 8:**
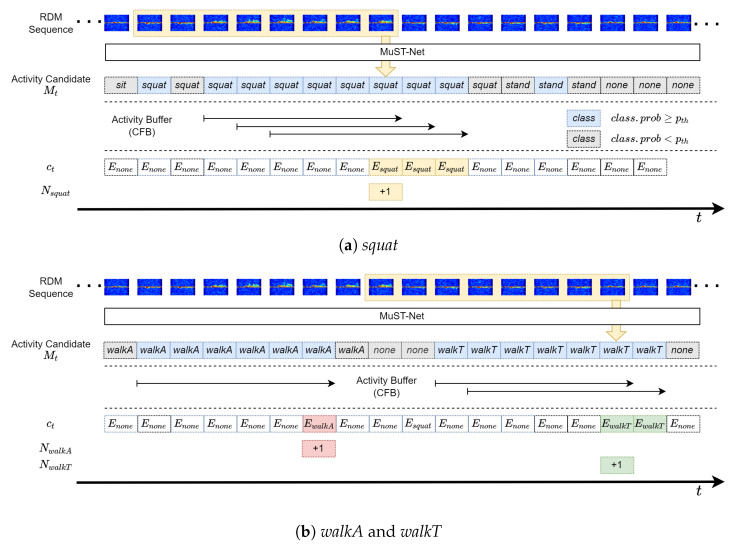
Activity detection and logging process.

To mitigate frame-level misclassifications and ensure consistent event-level outputs, we implement a composite activity-confirming framework for the MuST-Net output stream ($M_{t}$). This mechanism verifies that a predicted behavior (${\hat{y}}_{t}$) persists with high confidence ($p_{t}$) over a predefined temporal window. In addition, we propose an algorithm that systematically quantifies the frequency of different user behaviors. Unlike fixed-window [16] or stride-based [10] classifiers that produce only per-segment labels, this process detects activity boundaries in a live stream, suppresses boundary misclassifications, and counts event occurrences without double counting. The overall process is illustrated in Figure 8, with specific operations detailed in Algorithms 1 and 2.
**Algorithm 1** Activity Detection Algorithm
**Initialize:** $c_{prev}\leftarrow E_{none}$; $N_{a}\leftarrow 0$ for all activity classes *a*1:**function** ACTIVITY_DETECTION($M_{t}$)2:   push $M_{t}$ to CFB3:   $c_{t}\leftarrow Check\_Activity\left(CFB\right)$4:   **if** $c_{t}\neq E_{none}$ **and** $c_{t}\neq c_{prev}$ **then**5:   $N_{c_{t}}\leftarrow N_{c_{t}}+1$6:   **end if**7:   $c_{prev}\leftarrow c_{t}$8:**end function**

**Algorithm 2** Check Activity Function1:**function** Check_Activity(CFB)2:   class ← CFB[0].class3:   $detect\leftarrow (CFB\left[0\right].class.prob\geq p_{th})$4:   **for** n∈{1,2,…,W−1} **do**5:   detect ← $detect\times (CFB\left[n\right].class.prob\geq p_{th})$6:   **if** class≠CFB[n].class or detect=0 **then**7:      **return** $E_{none}$8:   **end if**9:   **end for**10:   **return** class11:
**end function**


#### 3.3.1. Activity Buffering

At the time points when an event starts or ends, the motion signature of the activity class with the highest probability received from MuST-Net may fail to exceed the threshold value. Unlike previous studies that prepared RDM data with fixed time lengths per motion unit for activity detection, this study aims to detect the start and end boundaries of defined activities from a continuous RDM stream received live, including intervals with no defined actions, and to measure the count of activities based on these boundaries. Therefore, for the output stream of motion signatures of defined classes from MuST-Net, a certain activity is considered detected when the motion signature of the activity class with the highest probability is sustained and its probability consistently exceeds the threshold value during a pre-configured time period.

**Figure 9:**
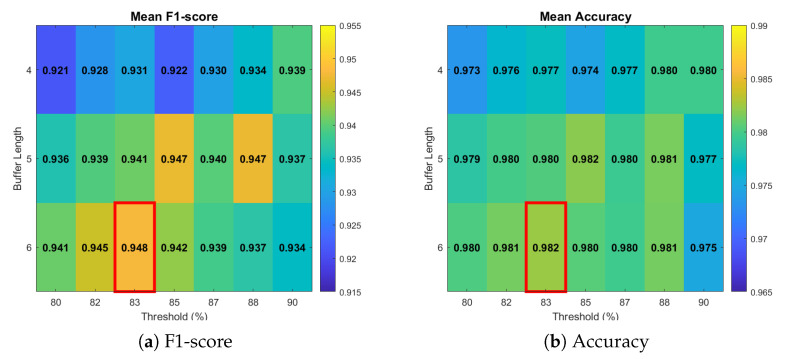
Sensitivity analysis for buffer length and threshold.

We maintain the six most recent motion signatures from MuST-Net in a consensus FIFO buffer (CFB). An activity is confirmed detected if all the highest-probability motion signatures for each frame in the buffer are of the same class and all the highest-probability signatures are over the pre-configured threshold, i.e., $p_{t}\geq p_{th}$. These values were selected based on a sensitivity analysis over W∈{4,5,6} and $p_{th}\in \left[0.80,0.90\right]$, evaluated on the full online detection sequences across all three sites and nine subjects. As shown in Figure 9, the combination of W=6 and $p_{th}=0.83$ achieved the highest macro F1 score and tied the best accuracy.

#### 3.3.2. Event Logging

For event logging, a subject’s indoor activities can be quantified by counting the number of their occurrences. The counter for each activity class is incremented based on the confirmed activity label returned by the activity buffer. Let $c_{t}$ denote the label returned by CHECK_ACTIVITY(CFB) at frame *t*, $c_{prev}$ denote the most recently confirmed label, and $N_{a}$ denote the cumulative occurrence count of activity class *a*. The counter is updated as

(3) $$N_{a}=\left\{\begin{array}{cc} N_{a}+1, & \mathrm{if}\;c_{t}=a,\;c_{t}\neq E_{none},\;\mathrm{and}\;c_{t}\neq c_{prev},\\ N_{a}, & \mathrm{otherwise}. \end{array}\right.$$

After each frame, $c_{prev}$ is updated to $c_{t}$ so that consecutive frames with the same confirmed label do not produce repeated increments. A new count is registered when $c_{t}$ corresponds to a defined activity ($c_{t}\neq E_{none}$) and differs from $c_{prev}$. Figure 8 shows examples of activity detection and counting for *squat*, *walkA* and *walkT* for W=6. Once six successive frames in the buffer share the same activity label with confidence above $p_{th}$, the corresponding event is counted, and the counter remains unchanged until the buffer transitions to a different state.

## 4. Experiment and Results

This section describes the experimental setup and environment for evaluating the MuST-Net-based HAR system. We present classification accuracy for the network alongside real-time detection results for the integrated system.

### 4.1. Experimental Setup

The experimental evaluation consists of two main parts. First, the performance of the MuST-Net activity classification is assessed in RDM input segments. Second, the accuracy of activity detection counts derived from the MuST-Net frame-wise activity classifier stream is evaluated.

For the first experiment, the data collection environment and the preparation of the dataset for training and testing are explained in Section 2. The MuST-Net model is optimized using a stochastic gradient descent optimizer, with the loss function using weighted cross-entropy, and is trained on an NVIDIA GeForce RTX 2080Ti GPU workstation. All experiments were executed using the PyTorch 2.5.1 framework.

For the second experiment, the proposed activity detection algorithm applied to the MuST-Net per-frame prediction stream is presented, given an online RDM input stream. Nine participants performed defined indoor activities and occasionally undefined motions in approximately 5 min trials, with the number of trials per subject varying by availability, yielding a total duration of 7972 s across three different environmental sites to ensure generalization. Sites A and B are laboratory spaces, while Site C is a lecture room, yielding a heterogeneous set of indoor environments. Across the three sites, the total floor area ranges from approximately 19.7 m^2^ to 27.4 m^2^, with internal obstacles (e.g., desks, chairs, shelves, and equipment) occupying approximately 5.3 m^2^ to 6.6 m^2^ per site and the placement of these obstacles differing across sites. The MuST-Net model used in all evaluations was trained solely on Site A data, making Sites B and C fully unseen test environments. Table 3 shows the information on the sequences played by the participants.

All human subjects participated in the experiments voluntarily and provided informed consent prior to data collection.

### 4.2. Performance Analysis of MuST-Net

#### 4.2.1. Channel Width Selection for Lightweight MuST-Net Deployment

To determine the optimal channel width for the interface between the 2D-CNN spatial feature extractor and the TCN temporal classifier in the proposed MuST-Net architecture, a comprehensive ablation study was conducted, as summarized in Table 4. The channel width determines the dimensionality of the spatial feature vector passed to the TCN and directly impacts both recognition performance and system resource utilization.

As shown in Table 4, balanced accuracy, macro-averaged precision, recall, and F1 score all increase as the channel width increases from 32 to 128, but additional gains become negligible for widths greater than 128. Specifically, expanding the channel width from 128 to 512 yields only marginal improvements—accuracy rises from 98.6% to 98.7%, and the macro F1 score remains virtually unchanged. Moreover, further increasing the width to 1024 results in reduced performance, indicating diminished generalization capacity and a likely optimization challenge for over-parameterized models under fixed training budgets.

From a computational point of view, the cost in GFLOPs and parameter size grows rapidly beyond 128 channels. The shift from 128 to 512 channels leads to a 6.75-fold increase in operations and a 12-fold increase in parameter count, without substantial improvement in detection metrics. Thus, channel widths greater than 128 offer minimal accuracy benefits while unnecessarily inflating resource consumption.

Taking into account the constraints of real-time embedded deployment and the goal of a compact, power-efficient system, a channel width of 128 was ultimately selected. This setting delivers state-of-the-art recognition performance while maintaining low computational and memory overhead, making it ideal for lightweight edge applications in practical indoor activity monitoring.

#### 4.2.2. Performance Evaluation of MuST-Net

**Figure 10:**
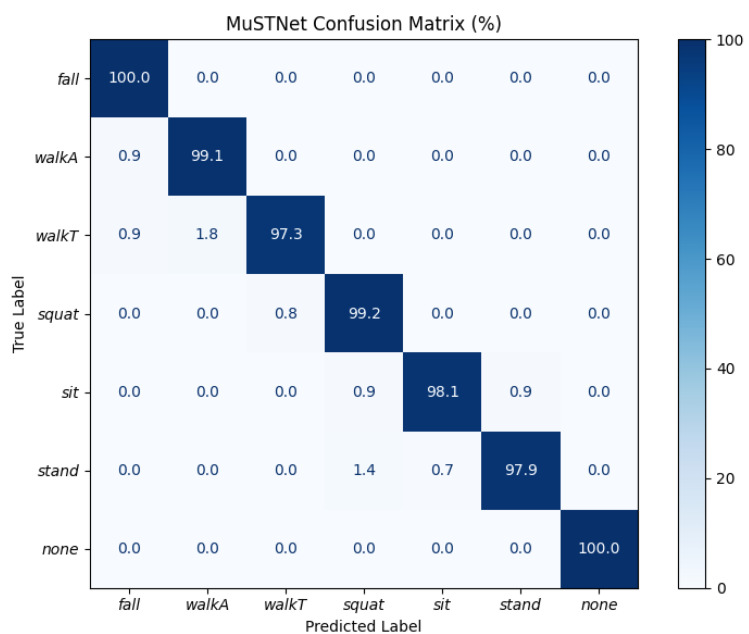
Confusion matrix on the test set.

Figure 10 presents the confusion matrix for the proposed MuST-Net architecture with a channel width of 128. This matrix summarizes the classification results for six indoor activities and *none*, providing a detailed view of the prediction accuracy and error distribution.

The diagonal dominance in the confusion matrix highlights the strong ability of MuST-Net to accurately classify all target activities. For the *fall* class, 387 of 387 samples were correctly classified, resulting in perfect recall. *Walk-away* also shows near-perfect performance, while *walk-toward* is occasionally misclassified as *walk-away*. *Stand* is the most frequently confused class, with a small fraction of samples misclassified as *squat*. The *none* class achieves 100% precision and recall, without misclassification.

Across all classes:

- The accuracy, precision, recall, and F1 score for activities are consistently near unity, with almost all errors attributed to subtle misclassifications between similar postures or transitional actions.
- The results confirm MuST-Net’s robustness, generalization capability, and temporal consistency for online indoor activity recognition, particularly with optimized channel width.
- Rare instances of misclassification (*stand* to *squat* and *walk-away* to *fall*) suggest the network’s high sensitivity to nuanced temporal or kinematic differences while also achieving lightweight deployment suited for embedded systems.

In summary, MuST-Net with a channel width of 128 achieves state-of-the-art reliable classification performance, validating the architecture’s suitability for real-time multi-class human activity monitoring in practical environments.

The results of comparisons with state-of-the-art classification networks are reported in Table 5. Compared to RDTNet for binary fall detection, expanding the label space from two to seven classes—a 3.5× increase—reduces the F1 score by only 0.8 percentage points, while accuracy and recall decrease by just 1.1 and 0.8 percentage points, respectively. Despite the larger number of classes and the need to distinguish closely related actions such as *squat* versus *sit* and *fall* versus *walk*, the performance drop remains on the order of a single percentage point. These results indicate that the proposed multi-class model effectively preserves the precision observed in the binary setting. The proposed MuST-Net achieves a comparable level of multi-class classification performance while substantially reducing computational cost and memory usage.

### 4.3. Online Activity Detection Results

This section comprehensively analyzes the recognition performance of the proposed online HAR system. In particular, in order to secure intra-class variability in static postures such as *sit* and *stand*, experiments were conducted by setting various directions and positions of subjects. Performance evaluation was conducted on nine subjects (S1–S9) across three distinct indoor environments (Sites A, B, and C), and detailed subject and dataset information is summarized in Table 3. Precision, recall, and F1 score were used as analysis indicators.

To evaluate the environmental generalizability of the HAR system proposed in this study, experiments were conducted at Sites A, B, and C, satisfying the indoor environmental conditions set in Section 2.1. Importantly, MuST-Net was trained exclusively on Site A data, making Sites B and C fully unseen test environments for assessment of cross-environment generalization. The overall performance of the proposed system is summarized in Table 6. As a result of the experiments, the system achieved a high accuracy of at least 0.97 across all classes, and for *fall* detection, the overall recall recorded a perfect value of 1.00, demonstrating the reliability of the system for safety-critical events. Most activities achieved an F1 score of 0.95 or higher, while the *sit* and *stand* classes recorded relatively lower F1 scores.

**Figure 11:**
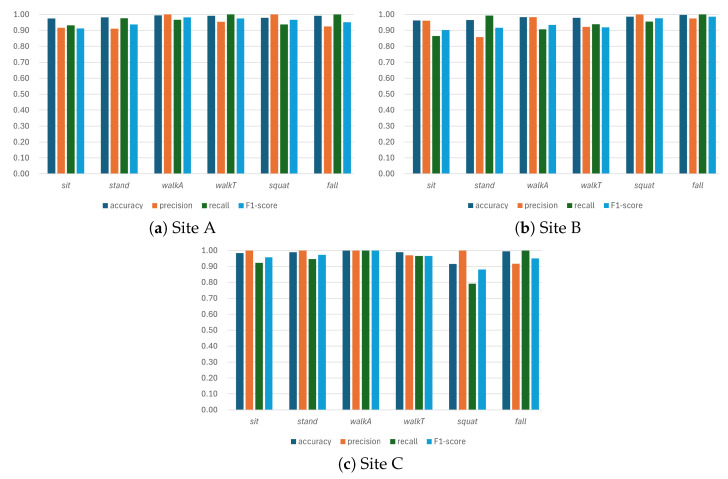
Comprehensive performance analysis of the proposed model across Sites A, B, and C.

The average F1 score by site is shown in Figure 11. The proposed model maintained high F1 scores across all three sites, although each site exhibited a distinct performance profile. Although Site A recorded a marginally higher overall F1 score than Sites B and C, the performance at Sites B and C—which were entirely unseen during training—remained at a comparable level, confirming that the system generalizes reliably across different indoor environments.

Looking at the activity-wise analysis in Figure 11, the lowest per-site F1 score varies depending on the environment. At Sites A and B, *sit* exhibited the lowest F1 score, and *stand* showed the lowest precision, indicating a relatively high false-positive rate at these two sites. This is interpreted as the result of the *stand* action having a shorter signal duration than other dynamic actions, resulting in undefined microscopic movements between actions being mistaken for *stand*. On the other hand, fewer false detections were observed for *sit* at these two sites, owing to its relatively longer duration, consistent with previous biomechanical findings that *sit-to-stand* and *stand-to-sit* transitions involve an extended deceleration phase for postural stability [25]. In contrast, at unseen Site C, the error pattern shifted: the precision of *sit* and *stand* rose to 1.00, and their F1 scores reached 0.96 and 0.97 respectively, while *squat*, instead, became the weakest class due to a drop in recall to 0.79. This site-specific behavior suggests that the distinct layout and subject motion patterns at Site C altered the rhythm of composite *squat* motions, leading to occasionally missed detections. Nevertheless, all activities at Site C retained an accuracy of 0.92 or higher, preserving the overall reliability of the system, even in this previously unseen environment.

**Figure 12:**
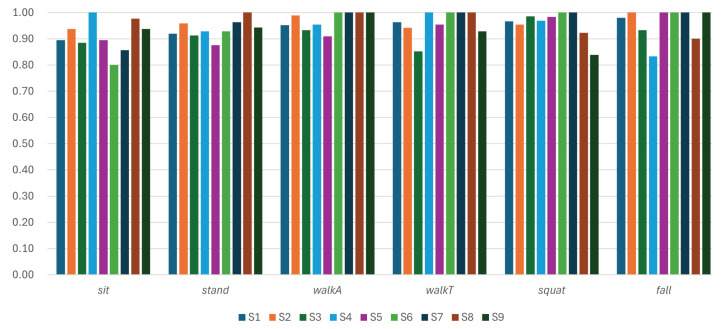
Performance across nine subjects in terms of F1 score.

Analyzing the effect of individual subject characteristics on performance (Figure 12), class-specific differences were identified, while a practical level of recognition performance was maintained for all nine subjects. The *walkA* and *fall* classes recorded the highest subject-averaged F1 scores, followed by *walkT* and *squat*, while *stand* and *sit* showed slightly lower averages. Notably, subjects S8 and S9—who were evaluated at Site C, an environment unseen during training—achieved F1 scores comparable to the overall average across most activity classes, providing evidence of the system’s cross-environment and cross-subject generalization capability.

Despite the overall good average performance, noticeable decreases in the F1 score were observed for specific subject–activity combinations. The most notable drop was S6’s *sit* F1 score of 0.80, which largely contributed to the lower overall *sit* F1 score observed at Site B. Additional notable drops include S4’s *fall*, S9’s *squat*, S3’s *walkT*, S7’s *sit*, S5’s *stand*, and S8’s *fall*. For S4’s *fall* case, the F1-score reduction reflects subject-specific false positives—other actions occasionally classified as *fall*—rather than missed detections, since the overall *fall* recall remained 1.00, preserving the safety-critical performance of the system. Likewise, S9’s lower *squat* F1 score corresponds to the recall drop observed at Site C, and S8’s lower *fall* F1 score reflects the slight precision drop for *fall* at the same unseen site. These performance fluctuations are due to inter-subject variability, since each subject’s walking speed, body tilt when seated, and behavioral habits introduce variations in reflected radar signals. Nevertheless, the consistently high F1 score maintained across all classes and subjects—including those at the unseen Sites B and C—demonstrates that the activity-buffer mechanism robustly handles both inter-subject motion variability and cross-environment distribution shifts.

### 4.4. Real-Time Indoor Behavior-Logging Application

To validate the practical utility of the proposed online activity detection algorithm, we developed a smartphone-based indoor behavior-logging application and conducted real-world experiments. The application employs the best-performing MuST-Net model deployed in a real-time system, allowing for live detection and logging of user activities in diverse indoor environments.

**Figure 13:**
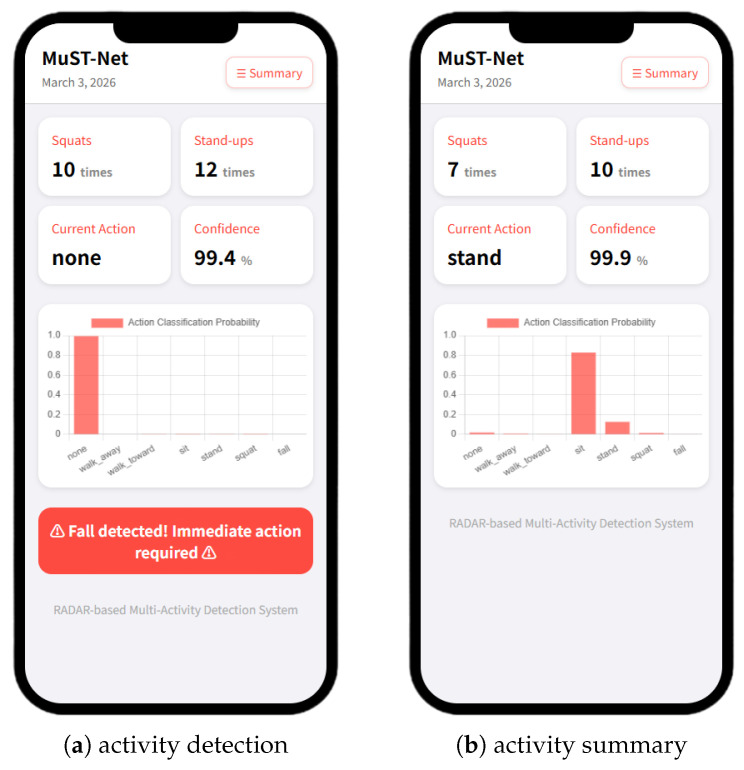
Real-time monitoring system application.

As described in Figure 13, the deployed system combines a radar sensing module with a FastAPI-based server and a mobile Web interface. The application continuously receives activity predictions from the detection algorithm, updates the recognized behavior class and its confidence level for each frame, and displays behavior statistics such as the number of squats and stand-ups detected over time. The interface refreshes every 100 ms, enabling users or operators to monitor current activities and receive immediate alerts—for example, in the event of a *fall*.

For rigorous evaluation, experiments were performed with new subjects and in settings differing from the training environment in terms of radar installation, obstacle arrangement, and action positioning. This generalization setup was designed to test the robustness of the system beyond the specific conditions seen during model training. The results confirmed that the application provides reliable and intuitive feedback on real-time human activities and facilitates practical deployment for continuous indoor behavior monitoring on a mobile platform.

## 5. Conclusions

This paper presented a robust indoor human activity recognition framework that uses mmWave FMCW radar for continuous motion analysis. The core contribution is MuST-Net, a lightweight hybrid architecture that combines a 2D CNN for spatial feature extraction and a TCN for temporal pattern modeling. This framework expands detection from binary *fall* classification to the simultaneous recognition of six distinct activities and a *none* state. To ensure stability in continuous monitoring, we introduced an activity-buffering process that employs an activity-confirming mechanism to suppress transient classification errors and maintain temporal continuity. This allows for a comprehensive count of indoor activities. MuST-Net, optimized with a 128-channel width for embedded deployment, achieved an overall classification accuracy of 98.6% on a large in-house dataset. The system demonstrated highly reliable recognition performance in all core activities, achieving 100% recall for *fall* detection and maintaining an accuracy of at least 97% across all classes, with F1 scores of at least 95% for most classes. Generalization was further validated across three distinct indoor environments and nine subjects, with two sites entirely unseen during training, confirming robustness against distribution shifts and strong cross-environment generalization for practical real-world deployments.

Future directions include expansion to more age groups reflecting elderly characteristics, multi-person detection, and robustness against various reflectors in real environments, toward broader use in smart homes and elderly monitoring.

## Figures and Tables

**Table 1:** Radar sensor configuration.

Parameter	Value
Number of Tx channels	1
Number of Rx channels	4
Center Freq.	78.4 GHz
Bandwidth	1.92 GHz
Chirp Slope	95 MHz/μs
Chirps per Frame	64
Frames per Sec	8
Range Bin	64
Doppler Bin	64
Maximum velocity	3.95 m/s
Maximum range	5 m

**Table 2:** Detailed architecture of CNN and TCN for MuST-Net.

(a) CNN				
Layer	Operation	Parameters		
		Ch.	Kernel	Grp.
0	Conv	8	3 × 3	1
	AvgPooling	8	4 × 4	1
1	Group Conv	8	3 × 3	8
	Point Conv	16	1 × 1	1
	MaxPooling	16	2 × 2	1
2	Group Conv	16	3 × 3	16
	Point Conv	32	1 × 1	1
	MaxPooling	32	2 × 2	1
3	Group Conv	32	3 × 3	32
	Point Conv	64	1 × 1	1
	MaxPooling	64	2 × 2	1
4	Group Conv	64	3 × 3	64
	Point Conv	128	1 × 1	1
	AvgPooling	128	2 × 2	1
(b) TCN				
Layer	Operation	Parameters		
		Ch.	Kernel	Grp.
0	Group Conv	128	4 × 1	128
	Point Conv	128	1 × 1	1
	MaxPooling	128	2 × 1	1
1	Group Conv	128	3 × 1	128
	Point Conv	128	1 × 1	1
	MaxPooling	128	2 × 1	1
2	Group Conv	128	3 × 1	128
	Point Conv	128	1 × 1	1
3	FC	256→32		
4	FC	32→7		

**Table 3:** Online sequences collected for indoor activity detection.

Subject	Site	Duration (s)	No. of Activities					
			*sit*	*stand*	*walkA*	*walkT*	*squat*	*fall*
S1	A/B	2198	46	53	37	39	92	16
S2	A/B	1524	28	33	30	31	62	12
S3	A/B	957	19	22	15	17	56	7
S4	A	593	8	8	13	14	32	5
S5	B	674	14	16	12	11	21	4
S6	B	327	12	13	8	8	5	3
S7	B	302	12	14	5	5	4	2
S8	C	742	19	19	13	15	28	4
S9	C	655	17	19	11	14	43	4
Total	–	7972	175	197	144	154	343	57

Sites A and B: laboratory; Site C: lecture room. Floor area: 19.7–27.4 m^2^ per site; obstacle coverage: 5.3–6.6 m^2^, with the layout differing across sites.

**Table 4:** Model performance metrics by channel width.

Metric	Channel Width					
	32	64	128	256	512	1024
Balanced Accuracy	0.974	0.978	0.986	0.982	0.987	0.981
Macro Avg. Precision	0.973	0.976	0.986	0.984	0.987	0.981
Macro Avg. Recall	0.974	0.978	0.986	0.982	0.987	0.981
Macro F1 Score	0.973	0.977	0.986	0.983	0.987	0.981
GFLOPs	0.003	0.007	0.016	0.039	0.108	0.332
Params (M)	0.008	0.022	0.065	0.217	0.782	2.961

**Table 5:** Comparison with state-of-the-art methods.

Metric	MuST-Net	RDTNet [12]	3DTSNet [13]	MFECNet [14]
Task Type	Multi	Binary	Multi	Multi
Performance				
Balanced Accuracy	0.986	**0.997**	0.980	0.996
Precision	0.986	0.994	n/a	**0.997**
Recall	0.986	0.994	n/a	0.994
F1 Score	0.986	0.994	n/a	0.995
Efficiency				
FLOPs (G)	**0.016**	0.041	1.020	0.252
Params (M)	**0.065**	0.200	80.600	2.501

**Table 6:** Overall performance of the proposed system.

Activity	Accuracy	Precision	Recall	F1 Score
*sit*	0.97	0.95	0.90	0.92
*stand*	0.98	0.90	0.98	0.93
*walkA*	0.99	0.99	0.95	0.97
*walkT*	0.99	0.94	0.97	0.95
*squat*	0.97	1.00	0.92	0.96
*fall*	0.99	0.94	1.00	0.97

## Data Availability

The data presented in this study are not available for sharing with third parties. Under the IRB-approved consent agreement, participants were informed that collected data would be used solely for the purposes of this study, with no provision for third-party access or secondary use.

## Abbreviations

The following abbreviations are used in this manuscript:

FMCW	Frequency-Modulated Continuous Wave
HAR	Human Activity Recognition
CNN	Convolutional Neural Network
TCN	Temporal Convolutional Network
RDM	Range–Doppler Map

## References

[1] Uddin M.Z., Soylu A. (2021). Human activity recognition using wearable sensors, discriminant analysis, and long short-term memory-based neural structured learning. Sci. Rep..

[2] Wei X., Wang Z. (2024). TCN-attention-HAR: Human activity recognition based on attention mechanism time convolutional network. Sci. Rep..

[3] Han H., Zeng H., Kuang L., Han X., Xue H. (2024). A human activity recognition method based on Vision Transformer. Sci. Rep..

[4] Huan S., Wang Z., Wang X., Wu L., Yang X., Huang H., Dai G.E. (2023). A lightweight hybrid vision transformer network for radar-based human activity recognition. Sci. Rep..

[5] Bouazizi M., Mora A.L., Feghoul K., Ohtsuki T. (2024). Activity Detection in Indoor Environments Using Multiple 2D Lidars. Sensors.

[6] Benedek C., Galai B., Nagy B., Janko Z. (2018). Lidar-Based Gait Analysis and Activity Recognition in a 4D Surveillance System. IEEE Trans. Circuits Syst. Video Technol..

[7] Mahmud S., Parikh V., Liang Q., Li K., Zhang R., Ajit A., Gunda V., Agarwal D., Guimbretiere F., Zhang C. (2024). ActSonic: Recognizing Everyday Activities from Inaudible Acoustic Wave Around the Body. arXiv.

[8] Watanabe H., Terada T., Tsukamoto M. (2017). Gesture Recognition Method Utilizing Ultrasonic Active Acoustic Sensing. J. Inf. Process..

[9] Yao Y., Liu C., Zhang H., Yan B., Jian P., Wang P., Du L., Chen X., Han B., Fang Z. (2022). Fall Detection System Using Millimeter-Wave Radar Based on Neural Network and Information Fusion. IEEE Internet Things J..

[10] Kittiyanpunya C., Chomdee P., Boonpoonga A., Torrungrueng D. (2023). Millimeter-Wave Radar-Based Elderly Fall Detection Fed by One-Dimensional Point Cloud and Doppler. IEEE Access.

[11] Li Z., Le Kernec J., Abbasi Q., Fioranelli F., Yang S., Romain O. (2023). Radar-based human activity recognition with adaptive thresholding towards resource constrained platforms. Sci. Rep..

[12] Yang L., Ye W. (2024). Design of a Two-Stage Continuous Fall Detection System Using Multiframe Radar Range–Doppler Maps. IEEE Sens. J..

[13] Li W., Wang W., Zhang D., Gegentuya (2023). Human motion recognition method using millimeter-wave radar based on 3DTSNet. 2023 5th International Conference on Electronics and Communication, Network and Computer Technology (ECNCT).

[14] Yuan X., Li J., Chen Q., Zou G. (2025). MFECNet: Multifeature Fusion Extra Convolutional Network Based on FMCW Radar for Human Activity Recognition. IEEE Trans. Instrum. Meas..

[15] Liang J., Huang Y., Huang Z. (2022). Fall Detection System Based on Millimeter Wave Radar and Machine Learning. 2022 6th International Conference on Robotics and Automation Sciences (ICRAS).

[16] Scherer M., Magno M., Erb J., Mayer P., Eggimann M., Benini L. (2021). TinyRadarNN: Combining Spatial and Temporal Convolutional Neural Networks for Embedded Gesture Recognition with Short Range Radars. IEEE Internet Things J..

[17] European Commission, Eurostat (2024). Housing in Europe—2024 Interactive Edition: Size of Housing. https://ec.europa.eu/eurostat/web/interactive-publications/housing-2024#size-of-housing.

[18] OECD (2024). Affordable Housing Database (AHD), Indicator HC2.1: Living Space—Definitions and Methodology. https://oe.cd/ahd.

[19] UN-Habitat (2021). SDG Indicator 11.1.1 Metadata: Proportion of Urban Population Living in Slums, Informal Settlements or Inadequate Housing. https://data.unhabitat.org/pages/guidance.

[20] Cao Z., Li Z., Guo X., Wang G. (2021). Towards Cross-Environment Human Activity Recognition Based on Radar Without Source Data. IEEE Trans. Veh. Technol..

[21] Li X., He Y., Zhang J.A., Jing X. (2021). Supervised Domain Adaptation for Few-Shot Radar-Based Human Activity Recognition. IEEE Sens. J..

[22] Wu G., Xue S. (2008). Portable Preimpact Fall Detector with Inertial Sensors. IEEE Trans. Neural Syst. Rehabil. Eng..

[23] Robinovitch S.N., Chiu J., Sandler R., Liu Q. (2000). Impact severity in self-initiated sits and falls associates with center-of-gravity excursion during descent. J. Biomech..

[24] Liu B., Hu X., Zhang Q., Fan Y., Li J., Zou R., Zhang M., Wang X., Wang J. (2016). Usual walking speed and all-cause mortality risk in older people: A systematic review and meta-analysis. Gait Posture.

[25] Lou S.-Z., You J.-Y., Tsai Y.-C., Chen Y.-C. (2021). Effects of Different Assistive Seats on Ability of Elderly in Sit-to-Stand and Back-to-Sit Movements. Healthcare.

